# Association of the serum uric acid to high-density lipoprotein cholesterol ratio with in-hospital mortality in patients with acute kidney injury: a retrospective cohort study

**DOI:** 10.3389/fendo.2026.1798688

**Published:** 2026-06-19

**Authors:** Desheng Zhang, Gen Li, Guohao Xu, Lijun Yin, Jianghua Guo, Jun Lv

**Affiliations:** Emergency Department, Shenzhen Nanshan People’s Hospital, Shenzhen, Guangdong, China

**Keywords:** acute kidney injury, HDL cholesterol, in-hospital mortality, prognostic marker, uric acid

## Abstract

**Background:**

Acute kidney injury (AKI) has a high in-hospital mortality rate, requiring better prognostic tools. The uric acid to HDL cholesterol ratio (UHR), a novel biomarker of metabolic inflammation, was investigated for predicting mortality in AKI patients.

**Methods:**

A retrospective cohort study was conducted from January 2021 to January 2024, including 500 adult AKI patients diagnosed according to KDIGO criteria. Patients with chronic kidney disease requiring dialysis, those transferred to other facilities, and those with incomplete medical records were excluded. Data on demographics, comorbidities, vital signs, laboratory tests, and clinical outcomes were extracted from electronic medical records. The primary outcome was all-cause in-hospital mortality.

**Results:**

The study enrolled 435 survivors and 65 non-survivors. Non-survivors were older and had a higher prevalence of hypertension and diabetes, with more severe renal impairment and inflammation, indicated by higher serum creatinine, blood urea nitrogen, and C-reactive protein levels. The univariate analysis revealed significant associations between in-hospital mortality and several variables, including UHR, age, serum creatinine, blood urea nitrogen, urine output, C-reactive protein, and estimated glomerular filtration rate. In multivariate analyses, with each unit increase in UHR associated with a 55% higher hazard of in-hospital mortality after adjustment (HR: 1.55; 95% CI: 1.18-2.01, p=0.002). The association was particularly stronger in older patients, those with higher serum creatinine, elevated CRP levels, and lower urine output.

**Conclusions:**

UHR independently predicts in-hospital mortality in AKI, supporting its use for risk stratification, though further mechanistic studies are needed.

## 1 Introduction

Acute kidney injury (AKI) is a common and serious clinical condition characterized by a sudden decline in kidney function, leading to the accumulation of waste products and dysregulation of fluid, electrolyte, and acid-base balance (1). AKI affects up to 20% of hospitalized patients and is associated with significant morbidity and mortality (2) (3). Despite advances in understanding its pathophysiology, effective treatment strategies for AKI remain limited, emphasizing the need for better risk stratification and prognostic indicators (4).

The uric acid/HDL cholesterol ratio (UHR) has recently emerged as a potential marker of cardiovascular risk and inflammation (5). Uric acid is a product of purine metabolism and has been implicated in oxidative stress and endothelial dysfunction (6). Conversely, high-density lipoprotein (HDL) is known for its protective cardiovascular properties, including reverse cholesterol transport and anti-inflammatory effects (7). An elevated UHR may reflect an imbalance between pro-oxidative and antioxidative forces, potentially contributing to adverse outcomes in various clinical settings (8).

Evidence suggests that both high uric acid levels and low HDL cholesterol levels are individually associated with increased mortality risk in patients with kidney disease (9) (10). However, the prognostic value of their combined effect, as captured by UHR, has not been thoroughly investigated in AKI. Understanding this association could offer new insights into the pathophysiology of AKI and aid in identifying patients at higher hazard of adverse outcomes.

This study aims to evaluate the association between UHR and in-hospital mortality among patients with AKI. By examining the prognostic significance of UHR, this study seeks to enhance risk stratification capabilities and potentially guide therapeutic interventions for this vulnerable population.

## 2 Methods

### 2.1 Study design and setting

This is a retrospective cohort study conducted at Shenzhen Nanshan People’s Hospital, a large tertiary referral hospital with approximately 1,500 inpatient beds in Shenzhen, China, from January 2021 to January 2024. The study aimed to evaluate the association between the serum uric acid to UHR and in-hospital mortality in patients with AKI).

### 2.2 Participants

The study population consisted of 500 patients diagnosed with AKI according to the Kidney Disease: Improving Global Outcomes (KDIGO) criteria. Inclusion criteria were: (1) adults aged ≥18 years, and (2) documented AKI during hospitalization. The specific diagnostic criteria used are summarized in **Supplementary Table 1**.

**Figure 1:**
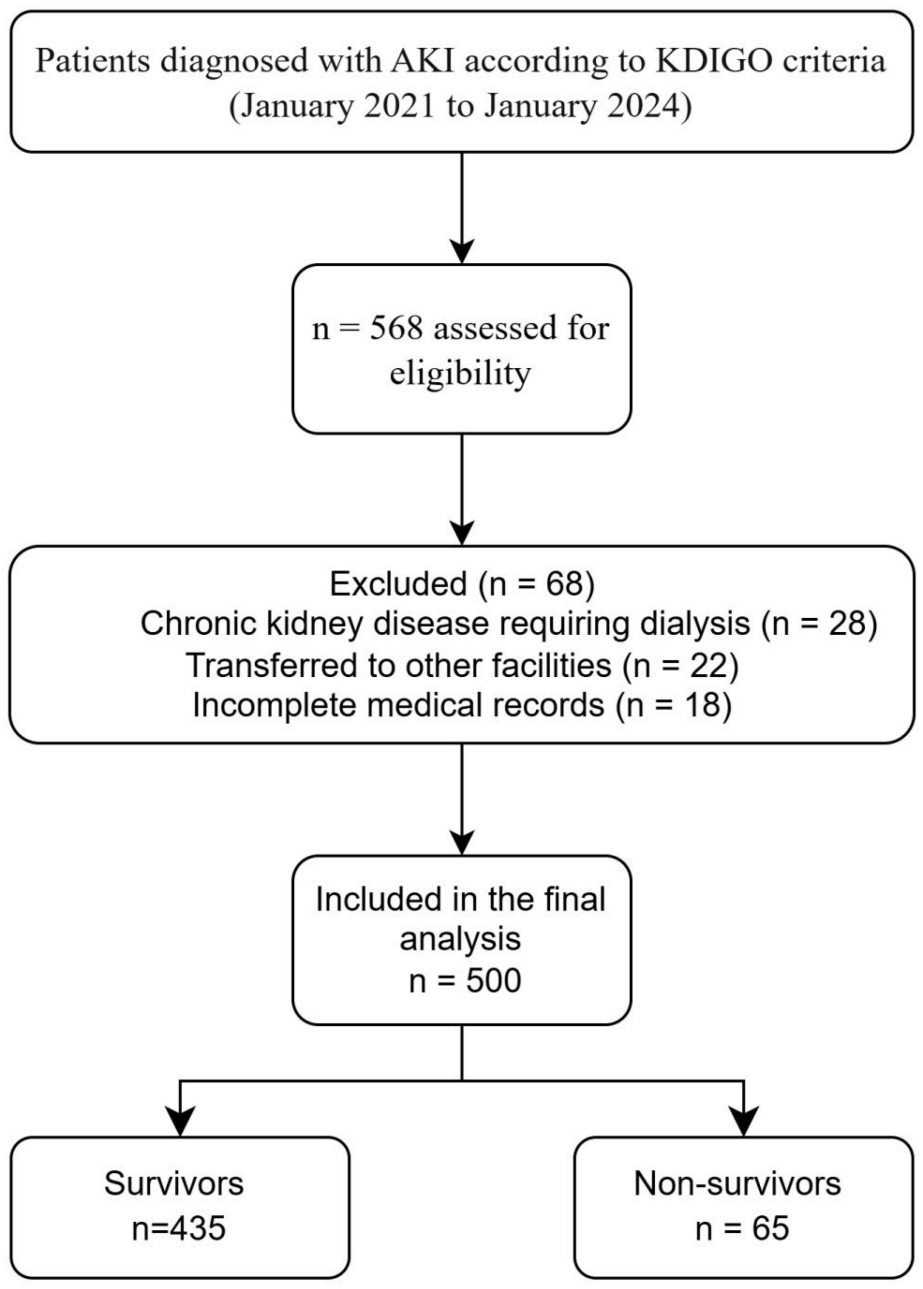
A flow diagram illustrating patient selection, inclusion, and exclusion.

Exclusion criteria included patients with chronic kidney disease requiring dialysis, those transferred to other facilities, and those with incomplete medical records. Patients with CKD on maintenance dialysis were excluded because the diagnosis and staging of superimposed AKI using standard KDIGO criteria (which rely on changes in serum creatinine and urine output) are challenging in this population due to altered baseline kidney function, variable residual renal function, and the confounding effects of regular dialysis sessions on laboratory parameters and fluid balance. Additionally, these patients often exhibit distinct pathophysiological features, including chronic uremia, persistent inflammation, and different metabolic profiles, which could confound the assessment of UHR as a prognostic marker specifically in acute kidney injury. The patient selection process is illustrated in **Figure 1**.

### 2.3 Data collection

Data were extracted from the hospital’s electronic medical records, including demographic information, comorbid conditions, vital signs, and laboratory tests. Blood tests, including serum uric acid and HDL cholesterol levels, were measured using standard laboratory techniques on a Roche Cobas c702 analyzer (Roche Diagnostics, Basel, Switzerland). Serum uric acid levels were determined using the uricase-peroxidase method, and HDL cholesterol levels were measured using a direct selective inhibitor method. The hospital laboratory reference ranges are summarized in **Supplementary Table 2**.

### 2.4 Variables and definitions

UHR was calculated as serum uric acid (mg/dL) divided by HDL cholesterol (mg/dL).

AKI was diagnosed according to the KDIGO criteria: an increase in serum creatinine by ≥0.3 mg/dL within 48 hours, or an increase to ≥1.5 times baseline within 7 days, or urine output <0.5 mL/kg/h for ≥6 hours. In-hospital mortality was defined as death from any cause during the index hospitalization.

### 2.5 Statistical analysis

Descriptive statistics were used to summarize baseline characteristics. Continuous variables were expressed as means ± standard deviations or medians with interquartile ranges, while categorical variables were summarized using counts and percentages. Differences between groups were assessed using t-tests or Mann-Whitney U tests for continuous variables and chi-square tests for categorical variables.

In-hospital mortality was analyzed as a time-to-event outcome using Cox proportional hazards regression models, with time defined as length of hospital stay (LOS) from admission until death (event) or discharge alive (censored). Both univariate and multivariate analyses were conducted. Variables significant at the 0.05 level in univariate analysis were included in the multivariate models. The proportional hazards assumption of the Cox regression model was tested using Schoenfeld residuals. The assumption was not violated for any of the variables included in the analysis (p > 0.05 for all variables), ensuring the validity of the model.

Subgroup analyses were performed by stratifying patients according to the median values of key continuous variables (age, serum creatinine, CRP, and urine output). The cutoff value of 0.16 for UHR in **Table 1** was chosen because it approximated the median UHR in the overall study cohort. As no established clinical cutoffs for UHR currently exist in the context of AKI, data-driven approaches (primarily medians) were used to ensure balanced subgroup sizes.

**Table 1:** Baseline characteristics by survival status.

Characteristic	Survivors (n=435)	Non-survivors (n=65)	p-value
Age, years	66.5 ± 15.4	74.2 ± 12.6	<0.001*
% >67 years (median cut-off)	48.7%	71.6%	<0.001*
Male, n (%)	240 (55.2%)	35 (53.8%)	0.82
Hypertension, n (%)	190 (43.7%)	39 (60.0%)	0.02*
Diabetes Mellitus, n (%)	132 (30.3%)	30 (46.2%)	0.01*
Serum Creatinine, mg/dL	2.8 ± 1.0	3.5 ± 1.2	<0.001*
% >3.2 mg/dL (median cut-off)	34.5%	59.9%	<0.001*
Blood Urea Nitrogen, mg/dL	30 ± 12	38 ± 15	<0.001*
Urine Output, mL	850 ± 320	500 ± 230	<0.001*
% ≤750 mL (median cut-off)	37.7%	86.1%	<0.001*
C-Reactive Protein, mg/L	8.5 ± 4.1	12.0 ± 5.7	<0.001*
% >9 mg/L (median cut-off)	45.1%	70.1%	<0.001*
eGFR, mL/min/1.73 m²	45 ± 18	32 ± 14	<0.001*
Uric Acid/HDL-C Ratio	0.13 ± 0.05	0.19 ± 0.06	<0.001*
% >0.16 (approximate median)	27.4%	69.1%	<0.001*
Length of hospital stay, days (median, IQR)	9 (6–14)	6 (3–10)	0.01*

UHR, uric acid to HDL cholesterol ratio; eGFR, estimated glomerular filtration rate; IQR, interquartile range; SD, standard deviation.

Continuous variables are presented as mean ± SD; categorical variables as n (%). Percentages above or below median values refer to the overall cohort. Significant differences (p < 0.05) are highlighted with *.

All statistical tests were two-sided, and a p-value <0.05 was considered statistically significant. Data analysis was conducted using R version 4.2.3.

## 3 Results

### 3.1 Baseline characteristics

The retrospective cohort study included 500 patients diagnosed with AKI, with 435 survivors and 65 non-survivors during their hospital stay. Baseline characteristics stratified by survival status are presented in **Table 1**. Notable differences were observed between survivors and non-survivors (p < 0.05 for multiple variables).

### 3.2 Association of plasma UHR with in-hospital mortality in patients with AKI

The univariate analysis revealed that the UHR, age, serum creatinine, BUN, urine output, CRP, and eGFR were significantly associated with in-hospital mortality. After adjusting for confounding variables in the multivariate analysis, UHR maintained its significant association with in-hospital mortality in patients with AKI. Specifically, each unit increase in UHR was associated with a 55% higher hazard of in-hospital mortality after adjustment (HR: 1.55; 95% CI: 1.18-2.01, p=0.002). Importantly, after full adjustment for age and other confounders in the multivariate Cox model, UHR remained independently associated with in-hospital mortality (adjusted HR 1.55, 95% CI 1.18–2.01, p=0.002), while age itself lost statistical significance (p=0.27). This demonstrates that the prognostic impact of UHR is not merely a reflection of older age. The results are summarized in **Table 2**. The proportional hazards assumption of the Cox regression model was tested using Schoenfeld residuals. The results confirmed that the assumption was not violated (p > 0.05 for all variables), ensuring the validity of the model.

**Table 2:** Cox regression analysis for in-hospital mortality.

Variable	Univariate HR (95% CI)	p-value	Multivariate HR (95% CI)	p-value
UHR	1.78 (1.32-2.42)	<0.001*	1.55 (1.18-2.01)	0.002*
Age (years)	1.04 (1.02-1.06)	<0.001*	1.03 (0.99-1.07)	0.27
Serum Creatinine (mg/dL)	1.22 (1.11-1.35)	<0.001*	1.15 (1.05-1.27)	0.003*
Blood Urea Nitrogen (mg/dL)	1.07 (1.03-1.12)	<0.001*	1.05 (1.01-1.10)	0.02*
Urine Output (per 100 mL decrease)	1.12 (1.05-1.18)	<0.001*	1.10 (1.04-1.17)	0.005*
C-Reactive Protein (mg/L)	1.09 (1.04-1.15)	<0.001*	1.07 (1.02-1.13)	0.02*
eGFR (mL/min/1.73 m²)	0.96 (0.94-0.98)	<0.001*	0.97 (0.94-1.02)	0.22

HR, hazard ratio; CI, confidence interval; UHR, uric acid to HDL cholesterol ratio; eGFR, estimated glomerular filtration rate.

Multivariate model adjusted for age, hypertension, diabetes mellitus, serum creatinine, blood urea nitrogen, urine output, C-reactive protein, and eGFR. Significant associations (p < 0.05) are highlighted with *.

### 3.3 Subgroup analysis

Among patients over 67 years old, UHR was significantly associated with a higher mortality risk (HR: 1.69, p<0.001) compared to those aged 67 or younger where the association was less pronounced (HR: 1.26, p=0.04).Although older age modified the strength of the association, UHR exerted an independent effect across age groups. Patients with higher serum creatinine levels (>3.2 mg/dL) exhibited a stronger link between UHR and mortality (HR: 1.75, p<0.001) than those with lower levels (HR: 1.30, p=0.02). The relationship between UHR and mortality was also more significant in patients with elevated CRP levels (>9 mg/L), showing an 80% increased risk (HR: 1.80, p<0.001). Conversely, this association was not significant in the subgroup with urine output above 750 mL, but it was significant in those with urine output at or below 750 mL, with a 65% increased risk (HR: 1.65, p<0.001).

In all, the association between the UHR and in-hospital mortality is particularly stronger in older patients, those with higher serum creatinine, elevated CRP levels, and lower urine output (**Table 3**).

**Table 3:** Subgroup analysis by median division.

Subgroup	HR for UHR (95% CI)	p-value
Age ≤ 67 years	1.26 (1.01-1.58)	0.04*
Age > 67 years	1.69 (1.25-2.29)	<0.001*
Serum Creatinine ≤ 3.2 mg/dL	1.30 (0.98-1.73)	0.25
Serum Creatinine > 3.2 mg/dL	1.75 (1.23-2.48)	0.02*
CRP ≤ 9 mg/L	1.20 (0.99-1.47)	0.20
CRP > 9 mg/L	1.80 (1.30-2.50)	<0.001*
Urine Output > 750 mL	1.18 (0.95-1.47)	0.35
Urine Output ≤ 750 mL	1.65 (1.21-2.26)	0.02*

HR, hazard ratio; CI, confidence interval; UHR, uric acid to HDL cholesterol ratio; CRP, C-reactive protein.

Subgroup analysis stratified by median values of key variables in the overall cohort (median age = 67 years, median serum creatinine = 3.2 mg/dL, median CRP = 9 mg/L, median urine output = 750 mL).Hazard ratios are from Cox regression models adjusted for the same covariates as in **Table 2**. P for interaction values were 0.03 for age, 0.04 for serum creatinine, 0.02 for CRP, and 0.03 for urine output. Significant associations (p < 0.05) are highlighted with *.

## 4 Discussion

The present study reveals a significant association between the UHR and in−hospital mortality in patients with AKI. Our findings underscore the potential of UHR as a prognostic marker in this context, aligning with the growing body of evidence that implicates both hyperuricemia and low HDL cholesterol in adverse outcomes in patients with kidney disease (11, 12). The present study reveals a significant association between the UHR and in−hospital mortality in patients with AKI. Our findings underscore the potential of UHR as a prognostic marker in this context, aligning with the growing body of evidence that implicates both hyperuricemia and low HDL cholesterol in adverse outcomes in patients with kidney disease (13). These results are consistent with previous studies showing an independent association between hyperuricemia and increased mortality in critically ill patients, including those with AKI (14), and in patients with chronic kidney disease (15). Moreover, low HDL cholesterol levels have been linked to poor outcomes in cardiovascular diseases, which share pathophysiological mechanisms with AKI given the close interplay between kidney and cardiovascular health (16).

The association between UHR and mortality may be explained by the role of uric acid in oxidative stress and inflammation, together with the protective effects of HDL cholesterol. Uric acid, a marker of oxidative stress, has been shown to predict the development and severity of AKI in various clinical settings (17) (18). HDL cholesterol, known for its anti−inflammatory and antioxidant properties, may mitigate the harmful effects of uric acid (19). Consequently, a low HDL cholesterol level could exacerbate the pro−oxidative state, tipping the balance toward increased inflammation and tissue damage in AKI (20). An elevated UHR could therefore reflect an imbalance between pro−oxidative and antioxidative forces, thereby contributing to adverse outcomes in AKI. Supporting this notion, hyperuricemia has been independently associated with increased mortality in chronic kidney disease, and low HDL cholesterol levels predict poor outcomes in cardiovascular conditions that share pathogenic pathways with AKI (15, 16).

In our subgroup analysis, the UHR–mortality link was influenced by age, renal function, inflammation and urine output. Older age, a known risk factor for AKI progression and mortality, may amplify the impact of UHR on outcomes (21). Likewise, higher serum creatinine and lower urine output, which indicate more severe kidney dysfunction, were associated with a stronger UHR–mortality relationship, suggesting that the prognostic value of UHR is greater in patients with more advanced AKI (22). Elevated CRP levels, a marker of systemic inflammation, also strengthened this association. This observation is consistent with the central role of inflammation in AKI pathogenesis and its link to worse outcomes (23, 24). Similar to other inflammatory indices, such as the granulocyte−to−lymphocyte ratio, which has been significantly associated with AKI after carotid stenting (25, 26), an elevated UHR in the setting of high CRP may denote a more severe inflammatory state and consequently a higher hazard of mortality (27).

One potential concern is that the observed association could be driven by older age, a well−established risk factor in AKI. However, our multivariate Cox regression models explicitly adjusted for age, and UHR retained independent significance (adjusted HR 1.55, p=0.002), whereas age became non−significant. Furthermore, subgroup analysis stratified by the median age demonstrated that the UHR–mortality link persisted in both younger (≤67 years) and older (>67 years) patients. These findings collectively rule out age as the sole driver of the observed effect and support UHR as an age−independent prognostic marker.

Our study adds to the existing literature by demonstrating the prognostic significance of UHR in AKI, a relationship that has not been thoroughly investigated previously. However, several limitations should be acknowledged. The retrospective, single−center design introduces the potential for selection bias; patients may reflect local referral patterns, socioeconomic characteristics and institutional treatment protocols that differ from other settings, and temporal changes in clinical practice during the study period may have further influenced patient selection. Due to the nature of the available data, important predictors of mortality in AKI—such as AKI etiology, KDIGO stage, presence of sepsis or septic shock, ICU admission, need for mechanical ventilation and vasopressor support—were not comprehensively captured. These factors are major drivers of in−hospital mortality and could potentially confound the observed association. Although we adjusted for multiple available covariates (age, sex, hypertension, diabetes, serum creatinine, BUN, urine output, CRP and eGFR), residual confounding cannot be excluded. Additional limitations include the relatively small sample size and the lack of mechanistic data such as gene expression or mRNA profiling. To address these concerns, we applied strict inclusion and exclusion criteria, adjusted for multiple confounders in multivariable Cox regression models, and performed subgroup analyses to ensure the robustness of our findings. While our study is observational and cannot establish causality or provide direct mechanistic insights, future prospective studies incorporating molecular analyses are warranted to elucidate the biological pathways linking uric acid/HDL cholesterol imbalance to inflammation, oxidative stress and AKI progression. Moreover, although our study population reflects a diverse group of AKI patients with varying demographics and comorbidities, future multi−center studies with larger, more diverse cohorts are needed to confirm the generalizability of these findings across different populations and healthcare systems.

## 5 Conclusion

In conclusion, our study demonstrates that the uric acid to HDL cholesterol ratio (UHR) is an independent predictor of in-hospital mortality in patients with acute kidney injury after multivariable adjustment. Subgroup analyses further indicate that this association is stronger among older patients and those with more severe renal dysfunction and systemic inflammation. These findings support the potential utility of UHR for risk stratification in AKI patients, although external validation in larger cohorts is warranted.

## Data Availability

The original contributions presented in the study are included in the article/**Supplementary Material**. Further inquiries can be directed to the corresponding author.
