# Supplementary material for: Association of the serum uric acid to high-density lipoprotein cholesterol ratio with in-hospital mortality in patients with acute kidney injury: a retrospective cohort study
Source: Front Endocrinol (Lausanne). 2026 Jun 19;17:1798688. doi: 10.3389/fendo.2026.1798688 (PMC13328003; doi:10.3389/fendo.2026.1798688)
Supplement: Supplementary file 1 [file Table1.docx]

**Table S1:** KDIGO Diagnostic Criteria for Acute Kidney Injury (AKI)

| Criterion | Definition |
| --- | --- |
| Serum Creatinine | Increase in serum creatinine by ≥ 0.3 mg/dL (≥ 26.5 µmol/L) within 48 hours OR Increase in serum creatinine to ≥ 1.5 times baseline within the prior 7 days |
| Urine Output | Urine output < 0.5 mL/kg/h for ≥ 6 hours |

**Note: AKI was defined by the presence of either serum creatinine or urine output criteria.**

**Table S2:** Reference Ranges for Key Laboratory Parameters

| Parameter | Reference Range | Unit |
| --- | --- | --- |
| Serum Uric Acid (Males) | 3.5–7.2 | mg/dL |
| Serum Uric Acid (Females) | 2.6–6.0 | mg/dL |
| HDL Cholesterol (Males) | >40 | mg/dL |
| HDL Cholesterol (Females) | >50 | mg/dL |

**Note: These reference ranges remained unchanged throughout the study period (January 2021 to January 2024).**
